# Energy compensation following consumption of sugar-reduced products: a randomized controlled trial

**DOI:** 10.1007/s00394-015-1028-5

**Published:** 2015-09-09

**Authors:** Oonagh Markey, Julia Le Jeune, Julie A. Lovegrove

**Affiliations:** 1 Hugh Sinclair Unit of Human Nutrition, Department of Food and Nutritional Sciences, University of Reading, Reading, Berkshire RG6 6AP UK; 2 Institute for Cardiovascular and Metabolic Research (ICMR), Department of Food and Nutritional Sciences, University of Reading, Reading, Berkshire RG6 6AP UK

**Keywords:** Sugar, Sugar-reduced products, Obesity, Body weight, Dietary energy compensation, Artificial sweeteners

## Abstract

**Purpose:**

Consumption of sugar-reformulated products (commercially available foods and beverages that have been reduced in sugar content through reformulation) is a potential strategy for lowering sugar intake at a population level. The impact of sugar-reformulated products on body weight, energy balance (EB) dynamics and cardiovascular disease risk indicators has yet to be established. The REFORMulated foods (REFORM) study examined the impact of an 8-week sugar-reformulated product exchange on body weight, EB dynamics, blood pressure, arterial stiffness, glycemia and lipemia.

**Methods:**

A randomized, controlled, double-blind, crossover dietary intervention study was performed with fifty healthy normal to overweight men and women (age 32.0 ± 9.8 year, BMI 23.5 ± 3.0 kg/m^2^) who were randomly assigned to consume either regular sugar or sugar-reduced foods and beverages for 8 weeks, separated by 4-week washout period. Body weight, energy intake (EI), energy expenditure and vascular markers were assessed at baseline and after both interventions.

**Results:**

We found that carbohydrate (*P* < 0.001), total sugars (*P* < 0.001) and non-milk extrinsic sugars (*P* < 0.001) (% EI) were lower, whereas fat (*P* = 0.001) and protein (*P* = 0.038) intakes (% EI) were higher on the sugar-reduced than the regular diet. No effects on body weight, blood pressure, arterial stiffness, fasting glycemia or lipemia were observed.

**Conclusions:**

Consumption of sugar-reduced products, as part of a blinded dietary exchange for an 8-week period, resulted in a significant reduction in sugar intake. Body weight did not change significantly, which we propose was due to energy compensation.

**Electronic supplementary material:**

The online version of this article (doi:10.1007/s00394-015-1028-5) contains supplementary material, which is available to authorized users.

## Introduction

There is much scientific debate regarding the potential impact of sugar consumption, especially in the form of sugar-sweetened beverages (SSB)^5^, on body weight status and various cardiometabolic health outcomes [1–5]. Specific policies on reducing over-consumption of energy and prevention of obesity and its comorbidities often include a recommendation to reduce sugar intake, as a means of reducing overall calorie intake and assisting with weight maintenance or prevention of weight gain [6, 7]. WHO guidelines recommend a population daily target for free sugars, namely ‘monosaccharides and disaccharides added to foods and beverages by the manufacturer, cook or consumer and the sugars naturally present in honey, syrups, fruit juices and fruit juice concentrates,’ of <10 % of total energy intake (% EI) [6]. A ‘conditional’ recommendation for a limit of <5 % EI for free sugars was also suggested, as although the desirable effects of adherence to limiting free sugar intake to this value outweigh the undesirable effects to public health. Policy makers will require substantial debate with involvement of various stakeholders before translation to an unconditional recommendation [6].

Until recently, it was advised that the UK population average intake of ‘non-milk extrinsic sugars’ (NMES) should provide no more than 10 % EI [8]. Data from the UK National Diet and Nutrition Survey (NDNS) highlight that population targets are currently being exceeded, with the mean NMES intakes of 11.5 and 14.9 % EI in adults aged 19–64 year and children aged 4–18 year, respectively [9]. In 2015, the Scientific Advisory Committee on Nutrition (SACN) recommended that the definition for ‘free sugars’ be adopted in the UK and advised that the population average intake of these sugars should not exceed 5 % EI [10]. The only minor difference between free sugars and NMES is that the latter definition includes half of the quantity of the sugars in stewed, dried or preserved fruit [11].

Consumption of commercially available sugar-reduced foods and beverages is seen as a potential strategy to lower sugar intake, and potentially EI [12]. Recently, the WHO have suggested that their 2015 guidelines on sugar intake should be used to develop a strategy for reformulation of processed foods, such as those rich in free sugars [6]. However, it should be recognized that the replacement of sugar-sweetened products with sugar-reduced or artificially sweetened alternatives may be associated with compensatory responses in EI and/or energy expenditure (EE), which in turn may only lead to modest weight loss [13–15]. Yet there are very limited data on the efficacy of using sugar-reformulated products on body weight, energy balance (EB) dynamics and the possibility of energy compensation [16].

The impact of sugar consumption on physiological markers of health is not fully established, but emerging evidence suggests that sugar consumption may be linked to lipid dysregulation, hypertension and inflammation [17, 18]. In short-term human intervention studies, sucrose supplementation has been shown to significantly increase total and low-density lipoprotein (LDL) cholesterol [19], as well as systolic and diastolic blood pressure (BP) [20] in overweight participants. However, more research is needed to examine the link between sugar consumption and novel and traditional markers of cardiometabolic health using a more physiologically relevant dietary model of sugar intake.

The reformulated foods (REFORM) study tested the hypothesis that, if energy compensation does not occur, reducing NMES intake to ≤10 % EI by exchanging reformulated, sugar-reduced foods and beverages for habitually consumed products would result in weight loss compared with matched regular products and that this would impact on markers of vascular health, glycemia and lipemia in healthy normal to overweight men and women. Indeed, preventative public health strategies that target healthy populations by addressing behavioral risk factors such as unhealthy dietary practices, including the high consumption of dietary sugars, could be effective approaches for promoting health and preventing cardiometabolic disease progression [21].

## Experimental methods

### Participants

The REFORM study included men (*n* = 16) and women (*n* = 34) aged 20–49 year who were not underweight or obese (BMI between 18.5 and 30 kg/m^2^) who were recruited from the local community of Reading, UK, in one cohort between March and September 2012. The intervention visits ran between May 2012 and March 2013. Recruitment strategies included targeted mailings to volunteers on local participant databases. Posters and flyers were circulated around the university campus as well as social and community groups in the Reading area. Key exclusion criteria were as follows: clinical diagnosis of diabetes, CVD, anti-inflammatory or hypertensive medication, smoking, excessive alcohol consumption (>21 units per week for men and >14 units per week for women), participation in regular vigorous exercise or fitness training (≥20 min × 3 times/week), pregnancy or lactating. The Joint British Societies’ JBS-2 total CVD risk calculator was used to estimate the probability (percentage chance) that a participant would experience a CVD event over the next 10-year period [22]. Dietary restraint was quantified using the Three-Factor Eating Questionnaire, with a score >11 (Factor I) indicating restrained eating [23, 24]; this score was used to characterize the cohort, but it was not used as an exclusion criteria for study participation.

Participants were not aware that the primary outcome of the study was body weight, as this could have influenced the study outcome. They were told that the purpose of the study was to examine the effect of reformulated fat, salt or sugar-reduced beverage and food items on risk factors for CVD. All procedures involving human participants were approved by the University of Reading’s Research Ethics Committee (12/03). Written informed consent was obtained from all participants before participation. The REFORM study was registered a clinical trial (Clinicaltrials.Gov ID: NCT01645995).

### Study design

The REFORM study was performed using a randomized, controlled, double-blind, crossover design and consisted of two 8-week dietary exchange intervention periods. Participants attended the Hugh Sinclair Unit of Human Nutrition on four separate occasions (before and after the two intervention phases). A computer-based minimization procedure (minimized on age, gender, BMI) was used to randomly allocate participants to consume either regular or sugar-reduced products (i.e., diet A or diet B) during their first 8-week dietary exchange period [25]. This was subsequently followed by a 4-week washout period after which the participants began the alternate study intervention.

Participants were advised prior to each visit to avoid strenuous physical activity (PA) and alcohol consumption for a 24-h period. Following a standardized low-fat evening meal (<400 kcal; <9 g fat), participants completed a 12-h fast.

### Dietary exchange model

For the REFORM dietary intervention, a flexible food exchange model, based on average UK food intake data of adults aged 19–64 year, was developed using a well-established approach that allows for minimal disruption to the habitual diet of free-living individuals [26]. The amount of exchangeable sugar in the free-living UK diet was calculated from the NDNS database [27] as the total NMES present in the following easily accessible food sources: breakfast cereals, baked beans, puddings, chocolate, sweet confectionary, sweet spreads, savory sauces, condiments, soft drinks, fruit juice, yoghurt, ice cream and other milks (Table 1). It was estimated that 60 % of the sugar-containing processed foods and beverages could be exchanged so as to manipulate the overall NMES composition of the habitual diet. Commercially available regular and sugar-reduced products, with a specific NMES profile, were tested in the dietary exchange model; it was quantified that the minimum daily NMES difference between the regular and reformulated dietary exchange periods was 32 g/day, with the reformulated arm consuming <7 % EI as NMES.

**Table 1:** REFORM food exchange model: removal of major exchangeable sources of dietary sugar in the UK diet and replacement of exchangeable sugar with REFORM study beverages and foods

	Mean quantity (g/day)	Energy (kcal/day)	CHO (g/day)	Total sugars (g/day)	NMES (g/day)	Starch (g/day)
NDNS intake^a^		1918	227.0	99.3	62.1	127.8
*Exchangeable sugar intake*						
Breakfast cereals	19	47	7.8	3.7	1.5	5.3
Puddings	12	19	2.3	1.5	1.3	0.4
Vegetables (baked beans)	93	42	9.1	3.4	1.5	4.6
Sugar, preserves and confectionary	24	100	15.9	18.2	13.9	0.0
Yoghurts, ice cream and other milks	38	58	4.5	3.0	2.4	0.3
Soft drinks and fruit juice	199	46	15.9	17.4	15.2	0.0
Savory sauces and condiments	22	38	2.3	4.7	1.5	3.0
Total exchangeable sugar intake		350	57.8	51.9	37.3	13.6
NDNS intake—total exchangeable sugar (Non-modifiable sugar intake)		1568	169	47.4	24.8	114.2
*Regular dietary exchange*						
Study beverage (mean of seven choices)^b^	315	160	37.7	37.6	37.7	0.1
Study food (mean of fifteen choices)^b^	74	107	18.5	14.7	12.6	3.8
Total intake	389	1835	225.2	99.7	75.1	118.1
*Reformulated dietary exchange*						
Study beverage (mean of seven choices)^b^	315	17	2.0	1.9	1.9	0.0
Study food (mean of fifteen choices)^b^	73	75	11.7	3.1	2.2	4.6
Total intake	388	1660	182.7	52.4	28.9	118.8
Mean difference between dietary exchanges		175	42.5	47.3	46.2	−0.7

*CHO* carbohydrate, *NMES* non-milk extrinsic sugars

^a^ Based on National Diet and Nutrition Survey (NDNS), 2003 for adults (19–64 year) [27]

^b^ Quantity varied depending on the study beverage or food. The replacement model was based on 1 beverage and 1 food per day and excluded *ad libitum* intake of condiments

### Dietary exchange intervention and compliance monitoring

For each 8-week dietary exchange period, participants exchanged ≥1 beverage and ≥1 food portion per day from their habitual diet with equivalent sugar-containing or sugar-reformulated products (for details of study foods, see Online Resource 1). After completing baseline study visits (at the beginning of each dietary assessment period), participants were given personalized advice about the dietary exchange intervention and how to incorporate the study products into their diet. Participants were given adequate study product supplies for a 4-week period at the beginning of each dietary assessment period. They also attended the Hugh Sinclair Unit of Human Nutrition for a food collection visit and dietary compliance assessment at week 4, the midway point in each dietary exchange period. During this visit, completed compliance sheets were assessed and any issues with dietary adherence were addressed and rectified. To encourage dietary adherence and reduce the likelihood of ‘product boredom’ [26], participants were provided with sufficient study products from a choice of 7 beverages including juice drinks and soft drinks, and 19 foods, including pasta sauce, baked beans, muesli, puddings and sweet confectionary (for details, see Online Resource 1). Participants were asked to replace habitually used sugar and condiments with those provided by the investigators ad libitum throughout the dietary exchange periods (Online Resource 1). Study product selection was based on each participants’ response to a food preference screening list that itemized the beverage, food, sugar and condiments options that were available to them as part of the dietary exchange intervention. They were asked to consume similar types of REFORM beverages/foods during their second arm of the study so that the regular and reformulated product types would be matched. Participants completed a daily dietary compliance sheet to assess their intake of the study products during the two 8-week dietary assessment periods (i.e., for a 56-day period). At the end of the first diet period, participants were asked to return unopened leftover products to the Nutrition Unit. They were instructed not to consume leftover study products during their washout period and were advised to return to their habitual diet.

Study products were presented to volunteers in a blinded manner. An investigator not involved with assessing study outcomes de-branded, relabeled or repackaged the study products so that they appeared identical between interventions, with the two dietary exchange products identifiable by diet A or B. The minimum beverage or food portion size was stated by weight and in general household measures.

### Assessment of intervention foods and blinding strategy

At the end of each 8-week dietary period, the study products were assessed for visual appeal, smell, taste, aftertaste and palatability using visual analogue scale (VAS) questionnaires [28]. Each VAS assessed a sensation on a 100-mm horizontal line, anchored at the beginning and end by opposing statements. On completion of the study, a retrospective questionnaire assessed participants’ perceived awareness of the order in which they had been assigned to the regular and reformulated study products.

### Assessment of dietary intake

Four-day weighed food diaries were completed prior to commencing and during the final week of the dietary exchange periods (week 0, 8, 12, 20). Participants completed their food diaries over three weekdays and one weekend day, with the same days repeated for subsequent food diaries. Detailed verbal and written instructions for completing their 4-day weighed food diaries were given to participants ≥7 days prior to their first study visit. A set of sample diaries and a set of digital scales were also provided, and the importance of not changing habitual dietary patterns was emphasized. Participants were asked to record recipes used during cooking and retain packaging from ready meals so that the items could be added to the dietary database. During study visits, food diaries were assessed for completeness. If necessary, further detail was collected to facilitate precise data entry.

Dietplan software (version 6.60; Forestfield Software Ltd.) was used to calculate specific energy and macronutrient intake. The nutrient composition of foods consumed was based on NDS Nutrient Database or McCance and Widdowson’s food tables. Dietary fiber intake was defined as non-starch polysaccharide (NSP) using the technique of Englyst and Cummings [29]. The composition of the study products was also added to Dietplan, based on manufacturer details.

### Assessment of physical activity

Participants were instructed to wear a triaxial accelerometer (GT3X + activity monitor; Actigraph, LLC) directly above the right iliac crest during sleeping and waking hours (except during water-based activities) for seven consecutive days, over the same time period that dietary intake was assessed. Device initialization, data processing and analysis were conducted using Actilife data analysis software (version 6.4.5). For analysis inclusion, participants were required to have produced counts on their activity monitor for ≥4 days (>600 min/day of wear time) [30]. Non-wear time was defined as ≥60 min of zero activity counts [31]. Data were summarized in 60-sec epochs, and mean EE from PA (EE_PA_) was calculated (kcal/day). Total daily step count, based on accelerometry, was also calculated and was used to estimate PA levels with <5000, 5000–7400, 7500–9999 and 10,000 steps/day categorizing participants as sedentary, low active, somewhat active and active, respectively [32, 33].

### Basal metabolic rate

Basal metabolic rate (BMR) for each participant was calculated based on age, gender and body weight using the Henry equation [34].

### Body weight

Upon arrival at the Nutrition Unit, height was measured to the nearest 0.1 cm using a wall-mounted stadiometer (first visit only). While wearing light garments, body mass (kg) and body fat (%) were assessed by Tanita Body Composition Analyzer (BC-418 MA; Ill, USA) using standard settings (normal adult body type and 1 kg for clothing) at week 0, 8, 12 and 20.

### Blood pressure and arterial stiffness

Participants rested semi-recumbent in a temperature-controlled vascular suite (22 ± 1 °C). After a 20-min rest and familiarization period, BP was recorded in triplicate using an ambulatory upper arm BP monitor (ABPM TM-243; A&D Instruments Ltd.). Digital volume pulse (DVP) and pulse wave analysis (PWA) were assessed as markers of vascular stiffness. DVP was determined by placing a photoplethysmographic transducer on the left index finger (Pulse Trace PCA 2 device; Micro Medical Ltd.). Measurements were taken in triplicate for calculation of mean reflection index (DVP–RI) and stiffness index (DVP–SI) [35]. PWA was carried out by a single trained operator using the SphygmoCor (ScanMed Medical; see [36] for details). The pulse pressure wave at the radial artery was recorded using applanation tonometry [37]. The mean of two values with the highest operator index (≥80) was averaged to calculate the augmentation index (AIx), a measure of overall systemic arterial stiffness and AIx adjusted to a standard HR of 75 bpm (AIx HR75).

### Blood sampling and biochemical analysis

Venous blood samples were collected and centrifuged at 3000 rpm for 15 min at 4 °C. Serum and plasma were separated and stored at −80 °C until subsequent analysis. Concentrations of serum lipids [total cholesterol, high-density lipoprotein cholesterol and triacylglycerol (TAG)], C-reactive protein, non-esterified fatty acid and glucose were determined using enzyme-based colorimetric tests on an ILab 600 Clinical Chemistry Analyzer (Instrumentation Laboratories Ltd.). The ratio of total:HDL cholesterol was calculated. LDL cholesterol concentrations were measured according to the Friedewald equation [38]. Plasma insulin concentrations were determined with enzyme immunoassay kits (Alere Ltd.). Samples from each participant were analyzed in a single run to minimize inter-batch variation.

### Energy intake and body weight simulation tool

For each participant, baseline variables including height, weight, age and gender were entered into the NIDDK human body weight simulation tool [16]. PA level, predicted from mean daily step count categories which were coded to match corresponding activity levels 1.4, 1.5, 1.6 and 1.9 in model [16, 33], was also entered. Collectively, these variables were used to provide a prediction of baseline EI (kcal/day).

For the body weight simulation, it was a required assumption that the PA level remained constant for the duration of each dietary exchange period. For body weight simulation, the value of 181 kcal/day was chosen to represent the mean energy deficit of the group during the reformulated dietary exchange period. This was calculated from the average of each participant’s 56-day caloric difference between the regular and reformulated dietary exchange periods (Online Resource 2). The energy deficit value was subtracted from the predicted baseline EI and predicted weight, after a 56-day body weight simulation was recorded. For body weight simulation following the regular dietary exchange period, it was assumed that EI remained constant, i.e., the same as that predicted at baseline.

### Power calculation

Power calculations were based on body weight, our primary outcome measure. An estimated weight loss of 1.2 kg was predicted with a daily conservative target difference of 181 kcal/day between the regular and reformulated dietary exchange phases for an 8-week period. The SD of weight reduction was estimated as 1.1 kg. Based on a previous calculation [39], an estimated recruitment of *n* = 37 participants was deemed necessary to give sufficient power to detect significant changes in our secondary outcome measures including biochemical analysis, if energy compensation did not occur, with *P* < 0.05 and 80 % power. With the allowance for a 25 % dropout rate, we aimed to recruit a minimum of 47 participants.

### Statistical analysis

All statistical analysis was performed on primary (body weight) and secondary outcome measures using SPSS (version 19.0; SPSS Inc.). Data that were not normally distributed, as assessed by the Shapiro–Wilk test, were logarithmically transformed. The potential impact of an intervention order was assessed by adding it as a main effect to our repeated measures *ANOVA* model. However, because no significant interactions between intervention order and our other main effects (time and diet) were detected, a two-way *ANOVA* was used to identify significant time *X* diet (*T* X *D*) interactions. Bonferroni post hoc corrections were applied to all data to control for multiple comparisons. For our primary outcome measure, multiple adjustments were made for age, gender and dietary restraint using a two-way *ANCOVA*. The association between observed and predicted (using the body weight simulation model [33]) body weights was analyzed using Pearson’s correlations. Paired *t* tests were used to compare dietary compliance data. Differences in baseline characteristics between participants randomly assigned to the regular and reformulated dietary exchange arms were assessed by using the independent *t* tests and the Chi square test for continuous and categorical variables, respectively (Online Resource 3). Data are presented as mean ± SD. *P* values < 0.05 were deemed to be significant.

## Results

### Participants

**Fig. 1:**
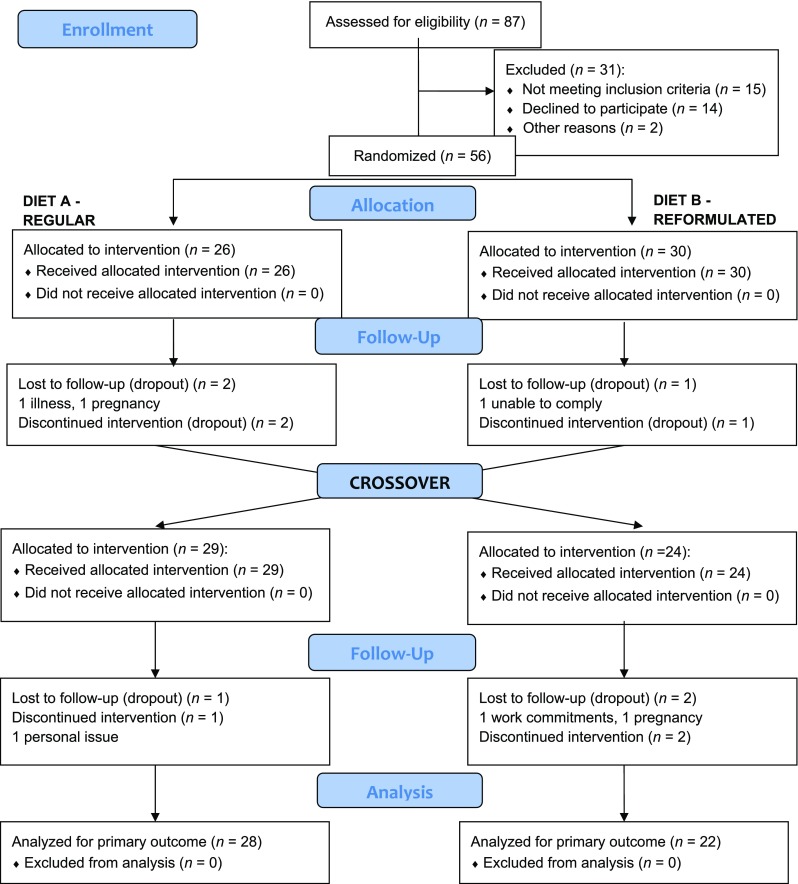
Flow of participants through the different stages of the REFORM study

Participant flow through the study is illustrated in Fig. 1. In total, 87 individuals were screened; 56 were recruited to the study and 50 participants (*n* = 40 White; *n* = 9 Asian; *n* = 1 Black) completed all four study visits. Both dietary exchange periods were well tolerated by participants, and no adverse events were reported. The baseline characteristics of the participants are highlighted in Table 2. Overall, participants had a 1.8 ± 3.4 % chance of developing CVD risk over the next 10-year period using the JBS-2 total CVD risk calculator [22]. Mean dietary restraint scores were 6.6 ± 4.2, with nine participants identified as restrained eaters. Five participants were excluded from the EI and EE_PA_ analysis due to insufficient data.

**Table 2:** Baseline characteristics of 50 participants who completed the intervention^a^

Parameter	
*Gender* [*n* (%)]	
M	16 (32)
F	34 (68)
Age (year)	31.6 ± 9.5
Body weight (kg)	69.8 ± 11.4
BMI (kg/m^2^)	24.0 ± 3.3
Body fat (%)	27.0 ± 9.6
Supine systolic blood pressure (mmHg)	116 ± 11
Supine diastolic blood pressure (mmHg)	71 ± 8
Glucose (mmol/L)	4.85 ± 0.42
Insulin (pmol/L)^b^	31.0 ± 14.3
Total cholesterol (mmol/L)	4.63 ± 0.71
HDL cholesterol (mmol/L)	1.50 ± 0.37
Total: HDL cholesterol ratio	3.22 ± 0.77
LDL cholesterol (mmol/L)	2.71 ± 0.61
Triacylglycerol (mmol/L)	0.93 ± 0.39
EI (kcal/day)^c^	2078 ± 675
EE_PA_ (kcal/day)^c^	446 ± 157
Mean accelerometer wear time (min/day)	1187 ± 128
Number of steps (counts/day)^c^	9064 ± 2615

*BMI* body mass index, *EE*_*PA*_ energy expenditure from physical activity assessed by triaxial accelerometry, *EI* energy intake, *HDL* high-density lipoprotein, *LDL* low-density lipoprotein

^a^ Values are presented as mean ± SD

^b^ *n* = 48

^c^ *n* = 45

### Energy and macronutrient intake

Mean habitual diets reported by the participants at baseline were comparable to representative dietary values for UK adults [9]. NMES intake fell to a mean of 8.3 % EI, meeting the criteria of ≤10 % EI on the sugar-reformulated diet. As a % EI, carbohydrate (*P* < 0.001), total sugars (*P* < 0.001) and NMES (*P* < 0.001) were lower, whereas fat (*P* = 0.001) and protein (*P* = 0.038) were higher on the sugar-reduced diet compared with the regular diet (Table 3).

**Table 3:** Daily energy and macronutrient intake at baseline (pre-) and during week-7 (post-) of the regular or reformulated dietary exchange period^a,b^

	Regular	Reformulated	*P* values		
			*T*	*D*	*T*X*D*
EI (kcal/day)			0.201	0.208	0.075
Pre-	1895 ± 568	1916 ± 553			
Post-	2049 ± 529	1887 ± 546			
Protein (% EI)^c^			0.383	<0.001	0.038
Pre-	16.4 ± 3.8	16.3 ± 3.8			
Post-	14.3 ± 2.6	15.4 ± 3.2			
Fat (% EI)			0.180	0.003	0.001
Pre-	33.9 ± 5.4	32.3 ± 6.5			
Post-	29.6 ± 6.1	32.8 ± 6.3			
CHO (% EI)			0.007	<0.001	<0.001
Pre-	47.3 ± 6.7	49.2 ± 6.9			
Post-	54.2 ± 7.3	48.2 ± 7.8			
Total sugars (% EI)^c^			0.000	0.314	<0.001
Pre-	20.6 ± 6.6	21.8 ± 7.3			
Post-	28.0 ± 7.0	17.1 ± 6.1			
Starch (% EI)			0.871	0.400	0.786
Pre-	25.6 ± 6.3	25.8 ± 6.2			
Post-	24.6 ± 5.6	27.1 ± 7.1			
NMES (% EI)			<0.001	0.003	<0.001
Pre-	10.5 ± 5.0	11.0 ± 6.2			
Post-	19.7 ± 5.6	8.3 ± 4.7			
Fiber intake (g/day)^c^			0.512	0.005	0.638
Pre-	15.0 ± 6.9	14.9 ± 6.5			
Post-	16.1 ± 6.1	16.6 ± 5.6			

*CHO* carbohydrate, *D* diet, *EI* energy intake, *NMES* non-milk extrinsic sugars, *T* time, *T*X*D* time X diet

^a^ Values are presented as mean ± SD

^b^ *n* = *45*

^c^ Data were log transformed

### Body weight and energy balance dynamics

There was no significant interaction for our primary outcome measure, body weight or percentage body fat, or for EI, EE_PA_ or EE_BMR_ (Table 4). Further analysis revealed that there was no significant impact of the intervention on body weight when it was adjusted for multiple covariates including age, gender and dietary restraint (time: *P* = 0.556; diet: *P* = 0.312; time *x* diet interaction: *P* = 0.203).

**Table 4:** Energy balance dynamics and body composition at baseline (pre-) and at 8 weeks (post-) after random assignment to a regular or reformulated dietary exchange period^a,b^

	Regular	Reformulated	*P* values		
			*T*	*D*	*T*X*D*
EI (kcal/day)			0.201	0.208	0.075
Pre-	1895 ± 568	1916 ± 553			
Post-	2049 ± 529	1887 ± 546			
EE_PA_ (kcal/day)			0.738	0.014	0.970
Pre-	432 ± 166	436 ± 169			
Post-	419 ± 201	405 ± 149			
EE_BMR_ (kcal/day)^e^			0.666	0.136	0.631
Pre-	1483 ± 206	1479 ± 210			
Post-	1489 ± 204	1489 ± 206			
Weight (kg)^c^			0.448	0.035	0.251
Pre-	69.8 ± 11.5	70.1 ± 11.3			
Post-	70.3 ± 11.3	70.2 ± 11.4			
BMI (kg/m^2^)^c,d^			0.300	0.401	0.202
Pre-	24.0 ± 3.4	24.1 ± 3.3			
Post-	24.1 ± 3.3	24.1 ± 3.4			
Body fat (%)^c^			0.222	0.447	0.541
Pre-	26.9 ± 9.6	27.2 ± 9.4			
Post-	27.1 ± 9.2	27.3 ± 9.4			

*BMI* body mass index, *D* diet, *EE*_*BMR*_ energy expenditure by basal metabolic rate, *EI* energy intake, *EE*_*PA*_ energy expenditure from physical activity assessed by triaxial accelerometry, *T* time, *T*X*D* time X diet

^a^ Values are presented as mean ± SD

^b^ *n* = 45

^c^ *n* = 50

^d^ Data were log transformed

^e^ Estimated with an equation including age, gender and body weight [34]

### Energy intake and body weight prediction model

Using the NIDDK mathematical model [16], predicted EI at baseline and post-intervention was calculated. The baseline predicted EI did not differ significantly between the regular and reformulated dietary exchange periods (Regular 2420 ± 385; Reformulated 2410 ± 389 kcal/day; *P* = 0.443). A mean of 79.2 and 80.8 % of predicted EI was reported from dietary analysis by participants prior to the regular and reformulated dietary exchange periods, but there was no significant difference in the baseline levels of under-reporting between the two dietary periods (*P* = 0.786). We observed a significant association between the predicted and observed body weight following the regular (*r* = 0.993, *P* < 0.001) and reformulated dietary exchange periods (*r* = 0.994, *P* < 0.001). The arithmetic difference between the predicted and observed body weight differed significantly following the 8-week regular and reformulated dietary exchange periods (Regular 0.9 ± 1.3 kg; Reformulated −1.5 ± 1.2 kg; *P* < 0.001).

### Blood pressure and arterial stiffness

There were no significant differences in systolic or diastolic BP between treatments (Table 5). Furthermore, no significant interactions were observed for DVP-SI, DVP-RI, AIx or AIx HR75 (Table 5).

**Table 5:** Blood pressure, arterial stiffness measurements and biochemical parameters at baseline (pre-) and at 8 weeks (post) after random assignment to a regular or reformulated dietary exchange period^a,b^

			*P* values		
Parameter	Regular	Reformulated	T	D	*T*X*D*
Supine systolic blood pressure (mmHg)^e^			0.813	0.188	0.518
Pre-	116 ± 11	116 ± 12			
Post-	116 ± 11	115 ± 12			
Supine diastolic blood pressure (mmHg)			0.113	0.970	0.844
Pre-	71 ± 7	72 ± 8			
Post-	71 ± 8	72 ± 8			
Digital volume pulse stiffness index (m/s)^c^			0.416	0.099	0.477
Pre-	5.5 ± 0.7	5.5 ± 0.6			
Post-	5.5 ± 0.6	5.6 ± 0.7			
Digital volume pulse reflection index (%)^c^			0.477	0.132	0.752
Pre-	55.5 ± 12.1	56.6 ± 11.6			
Post-	57.7 ± 10.7	58.1 ± 13.2			
Pulse wave analysis augmentation index (%)^e^			0.198	0.259	0.234
Pre-	13.9 ± 11.6	14.2 ± 13.1			
Post-	14.2 ± 11.7	15.1 ± 13.4			
Pulse wave analysis augmentation index adjusted to a standard HR of 75 bpm (%)			0.442	0.400	0.736
Pre-	6.3 ± 12.1	5.2 ± 14.2			
Post-	6.8 ± 12.3	7.3 ± 13.8			
Glucose (mmol/L)			0.611	0.010	0.220
Pre-	4.86 ± 0.40	4.84 ± 0.42			
Post-	4.90 ± 0.45	4.95 ± 0.39			
Insulin (pmol/L)^d,e^			0.185	0.072	0.665
Pre-	34.9 ± 20.4	33.6 ± 18.9			
Post-	37.6 ± 20.5	35.2 ± 20.4			
Total cholesterol (mmol/L)^e^			0.653	0.352	0.021
Pre-	4.67 ± 0.70	4.57 ± 0.69			
Post-	4.59 ± 0.65	4.58 ± 0.67			
HDL cholesterol (mmol/L)^e^			0.359	0.500	0.067
Pre-	1.54 ± 0.38	1.50 ± 0.37			
Post-	1.51 ± 0.38	1.51 ± 0.34			
Total:HDL cholesterol ratio^e^			0.672	0.993	0.457
Pre-	3.17 ± 0.76	3.17 ± 0.74			
Post-	3.18 ± 0.76	3.14 ± 0.70			
LDL cholesterol (mmol/L)^e^			0.338	0.387	0.360
Pre-	2.74 ± 0.61	2.66 ± 0.58			
Post-	2.67 ± 0.55	2.66 ± 0.56			
Triacylglycerol (mmol/L)^e^			0.954	0.460	0.419
Pre-	0.88 ± 0.37	0.90 ± 0.37			
Post-	0.92 ± 0.38	0.91 ± 0.41			
Non-esterified fatty acid (μmol/L)^e^			0.704	0.044	0.898
Pre-	464.5 ± 164.3	473.9 ± 176.2			
Post-	443.3 ± 189.7	436.5 ± 170.4			
C-reactive protein (mg/L)^e^			0.547	0.581	0.593
Pre-	0.93 ± 0.94	1.05 ± 1.35			
Post-	0.99 ± 1.03	1.21 ± 1.50			

*D* diet, *HDL* high-density lipoprotein, *LDL* low-density lipoprotein, *T* time, *T* X *D* time X diet

^a^ Values are presented as mean ± SD

^b^ *n* = 50

^c^ *n* = 47

^d^ *n* = 48

^e^ Data were log transformed

### Biochemical parameters

There were no significant changes in biochemical parameters following the regular compared with the reformulated products (Table 5).

### Assessment of dietary compliance, intervention foods and blinding strategy

The mean caloric and NMES differences between the regular and reformulated dietary exchange periods, inclusive of sugar and condiments, that were calculated for the two complete 8-week dietary assessment periods were 181 kcal/day and 54.4 g/day, respectively (see Online Resource 3 for dietary compliance data) with significantly higher intakes of NMES after the regular (Pre- 52.4 ± 35.8 g/day, Post- 98.9 ± 34.0 g/day), when compared with the reformulated arm (Pre- 54.3 ± 36.4 g/day, Post- 37.8 ± 19.9 g/day; *P* < 0.001). These values were higher than our target difference in NMES intake as calculated using the dietary exchange model (Table 1) and provide evidence that the dietary objectives of REFORM were successfully met and exceeded.

Lower mean ratings of taste (23.0 ± 10.8 vs 28.9 ± 11.6 mm; *P* < 0.001) and palatability (23.7 ± 11.2 vs 28.2 ± 12.5 mm; *P* = 0.009) of the reformulated compared with the regular products were observed. Visual appeal, smell and aftertaste of the products did not vary significantly between treatments (data not shown). A total of 83 % of all participants correctly identified the regular and reformulated products.

## Discussion

The REFORM randomized controlled trial is the first to examine the impact of sugar-reformulated food and beverage consumption on the dynamics of body weight, EB, BP, arterial stiffness, serum glucose and lipid concentrations in normal to overweight free-living adults [BMI mean (range) 23.5 (18.4–29.9) kg/m^2^] in a suitably powered study. We observed that when sugar-reduced foods and beverages were consumed as part of the habitual diet no significant change in body weight was observed. This was due to energy compensation; fat and protein intakes were both higher on the sugar-reduced diet, when compared to the regular diet.

Our cohort was relatively healthy with a generally low risk of developing CVD [22], yet they exceeded UK Department of Health dietary recommendations [8] by consuming >10 % EI from NMES at baseline. A strength of the REFORM study was the successful implementation of the novel dietary exchange model, resulting in the reduction in dietary targets of NMES, in line with recommendations [8] using both foods and beverages as intervention products. It is relatively simple to reduce the energy content of beverages by directly exchanging sugars with artificial sweeteners (AS). However, in more complex matrices, such as some of the reformulated foods used in our intervention, replacement of sugars with other nutrients such as starch is required to maintain functional properties of the products, and this may result in a smaller reduction in energy content [40]. Due to the inherent challenges of using sugar-reformulated foods in dietary intervention studies, their efficacy is poorly studied. Yet it is fundamental to determine their value as a strategy to reduce dietary sugar and energy for long-term weight control [12]. Specific dietary targets during the exchange periods of our intervention were largely achieved. Using the NIDDK simulation tool [16], body weight was predicted to be lower than observed, between the reformulated and regular dietary exchange periods, with no change in EE_PA_ and EE_BMR_, supporting our hypothesis of dietary energy compensation. Although the change did not reach statistical significance, EI was lower following the reformulated diet period, and this was matched by lower PA-related EE, suggesting that this may have also contributed to the observed energy compensation. Our study was limited by the fact that we did not use a calorimetric method for determination of EE [41, 42]; therefore, it is possible that small changes in total EE may have also contributed to energy compensation. During the regular arm of the study, it should be noted that participants increased their intake of NMES to 20 % EI, a 9 % increase when compared to baseline. In line with this, the studies of Reid and colleagues highlighted that, regardless of level of adiposity, when given in a blinded manner to free-living women for a 4-week period, SSB supplementation (4 × 250 mL; 430 kcal/day) was partially compensated for through voluntary reductions in macronutrient intake and hence did not lead to weight gain [33, 43, 44]. Following a 6-month intervention where overweight individuals were randomly assigned to consume either 1000 mL/day of sugar-sweetened cola, diet cola, semi-skimmed milk or water, Maersk et al. [45] did show that regular cola intake significantly enhanced fat accumulation in the liver, skeletal muscle and visceral fat, with no overall change in total fat or body weight. An increased production of plasma TAG was proposed as one plausible mechanism for the body weight-independent metabolic effect of dietary sugar [45]. Although we did not measure specific organ fat accumulation in the present study, it seems unlikely that we would have observed any changes in this parameter, given the lack of effect on circulating serum TAG.

Consumption of the sugar-reformulated products, in exchange for regular matched products in the REFORM study, did not result in differences in fasting BP, arterial stiffness, glycemia or lipemia. Despite not finding an impact of a 6-week high sucrose diet (25 % EI) on BP, PWA or any metabolic variable, Black and colleagues [19] observed modest increases in total and LDL cholesterol. In addition, during a 10-week sucrose supplementation period (~2 g/kg of body weight), where supplements were mostly given in the form of SSBs, Raben et al. [20] found a significant increase in systolic or diastolic BP compared to a diet rich in AS, while a more recent paper by this group showed that no independent effects on fasting insulin and postprandial TAG concentrations were observed, when data were adjusted for body weight change [46].

Although potential underlying mechanisms responsible for the relationship between added sugar consumption and cardiometabolic risk are not fully established, emerging research suggests that in addition to the indirect impact of weight gain on these parameters, excessive added sugar consumption may impact on several physiological pathways [47, 48]. When consumed in excess (>20 % EI), fructose intake has been adversely linked to de novo lipogenesis, BP, visceral and ectopic fat deposition, insulin sensitivity as well as fat oxidation [18, 45, 49], although these levels far exceed current intakes. Furthermore, men and women with a genetic predisposition to obesity had a more pronounced susceptibility to the impact of SSBs on adiposity [50]. Future investigation is necessary to evaluate the differential impact of ethnicity on adiposity and cardiometabolic risk markers in response to regular sugar and sugar-reduced product consumption.

There are possible limitations of the REFORM study. Although sufficiently powered to detect predicted weight changes associated with the energy deficit if EB was maintained, our intervention period may not have been adequate to identify changes in some secondary outcome measures, including arterial stiffness [51]. Additionally, our dietary exchange provided a realistic dietary sugar intake from consumption of commercially available sugar-reformulated foods and beverages, but this resulted in modest differences in sugar intake compared with other studies [18, 20, 33, 43, 44]. Despite this, the body simulation tool [16] predicted that these modest differences in dietary sugar intake were sufficient to detect a difference of 2.4 ± 1.7 kg in body weight between the diets. This was not observed and indicates partial compensation for the lower or higher EI during the reformulated or regular dietary arm, respectively, confirming our dietary intake data. We also acknowledge the limitation of estimating EI using self-reported measurements [41], but note that this issue was deemed to be less significant, given that we supplied the regular and sugar-reduced study foods for the participants to consume during their dietary exchange periods and monitored dietary compliance. Furthermore, mean EI predicted by the NIDDK model [33] did not differ between the regular and reformulated dietary exchange periods at baseline.

Evaluation of the efficacy of public health strategies for health promotion is essential. Previous food reformulation strategies targeting salt and *trans* fatty acid reduction have proven effective [7, 52], while the success of sugar-reformulated products is less clear. Systematic reduction in the sugar content of food stuffs, without any AS substitution, may be a feasible approach to reducing sugar consumption and re-educating the palate to accept lower sweetness [53, 54], while limiting the potential for consumers to subconsciously overcompensate for perceived ‘caloric savings’ attained by AS usage [55]. Indeed, the American Heart Association and American Diabetes Association state that the potential benefits of AS will not be appreciated if there is compensatory intake of energy from other macronutrient sources [56]. In an attempt to overcome the impact of psychological cues on EI and body weight, a double-blind study design was used in REFORM. However, in contrast to a previous study [57], the majority of participants correctly identified the regular and reformulated foods, due to the significant lower reported taste and palatability of the reformulated products. Moreover, our consumer acceptance findings indicate that significant improvements to the sensory qualities of a selection of sugar-reduced products, that were included in the REFORM intervention, are necessary [58]. This may have influenced conscious energy compensation and highlights the challenge faced by food industry in matching these sensory attributes in artificially sweetened foods and beverages.

In conclusion, consumption of sugar-reformulated foods and beverages as part of a blinded dietary exchange, resulted in a significant reduction in sugar intake, but had no significant effect on body weight, BP, fasting serum glucose or lipid concentrations, which was in part due to energy balance compensation. Future work is required to determine whether these findings have relevance to energy compensation dynamics in obese populations or fat accumulation in healthy populations over a long-term dietary intervention period.

## Electronic supplementary material

Below is the link to the electronic supplementary material.

Supplementary material 1 (DOCX 18 kb)

Supplementary material 2 (DOCX 20 kb)

Supplementary material 3 (DOCX 22 kb)
