# Supplementary material for: Energy compensation following consumption of sugar-reduced products: a randomized controlled trial
Source: Eur J Nutr. 2015 Sep 9;55(6):2137–49. doi: 10.1007/s00394-015-1028-5 (PMC5009173; doi:10.1007/s00394-015-1028-5)
Supplement: Supplementary file 1 — Supplementary material 1 (DOCX 18 kb) [file 394_2015_1028_MOESM1_ESM.docx]

| **Electronic Supplementary Material**  Energy compensation following consumption of sugar-reduced products: a randomized controlled trial, European Journal of Nutrition, Oonagh Markey, Julia Le Jeune and Julie A. Lovegrove, University of Reading, [j.a.lovegrove@reading.ac.uk](mailto:j.a.lovegrove@reading.ac.uk)  **Online Resource 1** Details of the REFORM study beverage and food portion sizes during the regular and reformulated dietary exchange periods^a^ | | |
| --- | --- | --- |
| REFORM Study Items | Portion sizes | |
|  | Regular | Reformulated |
| Beverages |  |  |
| Soft drink (cola, lemonade, orange pop) | 330 mL | 330 mL |
| Cranberry & raspberry juice drink | 333 mL | 333 mL |
| Blackcurrant juice drink (concentrate) | 80 mL | 80 mL |
| Fruit squash concentrate (summer fruit, orange) | 400 mL | 400 mL |
| Foods |  |  |
| Jellies | 25 g | 25 g |
| Mints | 20 g | 20 g |
| Marshmallows | 20 g | 20 g |
| Chocolate (milk, dark, hazelnut) | 25 g | 25 g |
| Jelly (strawberry, orange, raspberry) | 125 g | 115 g |
| Vanilla ice-cream | 100 g | 100 g |
| Whipped dessert topping (as prepared) | 125 g | 125 g |
| Custard (as prepared) | 130 g | 130 g |
| Pasta sauce | 125 g | 125 g |
| Sweet and sour sauce | 125 g | 125 g |
| Baked beans | 200 g | 200 g |
| Fruit flavored yoghurt | 112 g | 112 g |
| Hot chocolate powder | 20 g | 20 g |
| Cappuccino powder | 18 g | 16 g |
| Muesli | 45 g | 45 g |
| Condiments |  |  |
| Sugar | Condiments were used *ad libitum* during the dietary exchange periods | |
| Chocolate spread  Tomato ketchup  Brown sauce  Preserve (raspberry, strawberry, marmalade) |  |  |
| ^a^Participants exchanged a minimum of one beverage and one food portion per day from their usual diet with equivalent sugar-containing (regular) or sugar-reduced (reformulated) products offered by the study. | | |
