# Supplementary material for: Energy compensation following consumption of sugar-reduced products: a randomized controlled trial
Source: Eur J Nutr. 2015 Sep 9;55(6):2137–49. doi: 10.1007/s00394-015-1028-5 (PMC5009173; doi:10.1007/s00394-015-1028-5)
Supplement: Supplementary file 2 — Supplementary material 2 (DOCX 20 kb) [file 394_2015_1028_MOESM2_ESM.docx]

| \| **Electronic Supplementary Material**  Energy compensation following consumption of sugar-reduced products: a randomized controlled trial,  European Journal of Nutrition, Oonagh Markey, Julia Le Jeune and Julie A. Lovegrove, University of Reading,  j.a.lovegrove@reading.ac.uk \| \| \| \| \| \| \| \| \| \| \| --- \| --- \| --- \| --- \| --- \| --- \| --- \| --- \| --- \| --- \| \| **Online Resource 2** Daily energy and macronutrient intake from study products during the regular and reformulated 56-day dietary exchange periods according to dietary compliance sheets^a^ \| \| \| \| \| \| \| \|  \| Regular \| Reformulated \| \| *P* value \| \| \| \| EI (kcal/d)^b^ \| 361 ± 102 \| \| 180 ± 83 \| \| < 0.001 \| \| Protein (g/d) \| 4.5 ± 2.1 \| \| 4.5 ± 2.4 \| \| 0.573 \|  \| \| \| Fat (g/d)^b^ \| 4.6 ± 2.4 \| \| 4.5 ± 2.4 \| \| 0.847 \|  \| \| \| Carbohydrate (g/d)^b^ \| 75.1 ± 19.9 \| \| 30.0 ± 13.2 \| \| < 0.001 \|  \| \| \| Total sugars (g/d)^b^ \| 66.0 ± 17.3 \| \| 10.2 ± 4.8 \| \| < 0.001 \|  \| \| \| NMES (g/d)^b^ \| 62.0 ± 15.9 \| \| 7.7 ± 4.0 \| \| < 0.001 \|  \| \| \| Starch (g/d)^b^ \| 9.7 ± 5.9 \| \| 10.6 ± 6.6 \| \| 0.059 \|  \| \| \| NSP (g/d) \| 3.2 ± 1.8 \| \| 3.4 ± 1.9 \| \| 0.007 \|  \| \| \| *EI Energy intake, NMES non-milk extrinsic sugars, NSP* non-starch polysaccharide (defined using the technique of Englyst and Cummings[_ENREF_1](#_ENREF_1) [[29](#_ENREF_29)])  ^a^Values are presented as mean ± SD. ^b^Data were log transformed. \| \| \| \| \| \|  \| \| \| |
| --- | --- | --- | --- | --- | --- | --- | --- | --- | --- | --- | --- | --- | --- | --- | --- | --- | --- | --- | --- | --- | --- | --- | --- | --- | --- | --- | --- | --- | --- | --- | --- | --- | --- | --- | --- | --- | --- | --- | --- | --- | --- | --- | --- | --- | --- | --- | --- | --- | --- | --- | --- | --- | --- | --- | --- | --- | --- | --- | --- | --- | --- | --- | --- | --- | --- | --- | --- | --- | --- | --- | --- | --- | --- | --- | --- | --- | --- | --- | --- | --- | --- | --- | --- | --- | --- | --- | --- | --- | --- | --- | --- | --- | --- | --- | --- |
