# Supplementary material for: Energy compensation following consumption of sugar-reduced products: a randomized controlled trial
Source: Eur J Nutr. 2015 Sep 9;55(6):2137–49. doi: 10.1007/s00394-015-1028-5 (PMC5009173; doi:10.1007/s00394-015-1028-5)
Supplement: Supplementary file 3 — Supplementary material 3 (DOCX 22 kb) [file 394_2015_1028_MOESM3_ESM.docx]

| **Electronic Supplementary Material**  Energy compensation following consumption of sugar-reduced products: a randomized controlled trial,  European Journal of Nutrition, Oonagh Markey, Julia Le Jeune and Julie A. Lovegrove, University of Reading, j.a.lovegrove@reading.ac.uk | | | |
| --- | --- | --- | --- |
| **Online Resource 3** Baseline characteristics for participants’ based on the order of allocation to the regular and reformulated dietary exchange arms *^a^* | | | |
| Parameter | Order 1^b^  (n = 22) | Order 2^c^  (n = 28) | *P* value |
| Gender, (n (%)) |  |  |  |
| M | 7 (32) | 9 (32) | 0.981 |
| Age (year)^8^ | 31.6 ± 10.2 | 31.1 ± 9.2 | 0.93 |
| Body weight (kg) | 70.5 ± 13.1 | 69.3 ± 10.1 | 0.71 |
| BMI (kg/m^2^) ^h^ | 24.2 ± 3.3 | 23.9 ± 3.4 | 0.82 |
| Body fat (%) | 25.9 ± 9.8 | 27.9 ± 9.6 | 0.47 |
| Supine systolic blood pressure (mm Hg) ^h^ | 116 ± 13 | 115 ± 11 | 0.80 |
| Supine diastolic blood pressure (mm Hg) | 71 ± 8 | 71 ± 8 | 0.82 |
| Serum glucose (mmol/L) | 4.95 ± 0.46 | 4.77 ± 0.37 | 0.15 |
| Serum total cholesterol (mmol/L) ^h^ | 4.78 ± 0.72 | 4.52 ± 0.69 | 0.17 |
| Serum HDL cholesterol (mmol/L) ^h^ | 1.46 ± 0.32 | 1.53 ± 0.41 | 0.58 |
| Serum Total:HDL cholesterol ratio | 3.42 ± 0.95 | 3.06 ± 0.57 | 0.10 |
| Serum LDL cholesterol (mmol/L) | 2.86 ± 0.71 | 2.59 ± 0.49 | 0.11 |
| Serum Triacylglycerol (mmol/L) ^h^ | 0.99 ± 0.43 | 0.88 ± 0.36 | 0.30 |
| Plasma insulin (pmol/L) ^h^ | 33.96 ± 16.09^d^ | 29.38 ± 14.68^e^ | 0.27 |
| EI (kcal/day) | 1926 ± 642^f^ | 1957 ± 590^g^ | 0.87 |
| EE_PA_ (kcal/day) | 460 ± 137^f^ | 442 ± 178^g^ | 0.55 |
| Mean accelerometer wear time (min/day)^h^ | 1215 ± 95^f^ | 1164 ± 148^g^ | 0.19 |
| Number of steps (counts/day) | 8996 ± 2789^f^ | 9118 ± 2523^g^ | 0.88 |
| *BMI* body mass index, *EE_P_*_A_ energy expenditure from physical activity assessed by tri-axial acclerometry, *EI* energy intake, *HDL* high density lipoprotein, *LDL* low density lipoprotein.  ^a^Values are presented as mean ± SD. Differences in baseline characteristics between participants randomly assigned to the regular and reformulated dietary exchange arms were assessed by using the independent t-tests and the chi-square test for continuous and categorical variables, respectively. ^b^Order 1= participants were randomly allocated to consume regular products during their first dietary exchange period. ^c^Order 2= participants were randomly allocated to consume reformulated products during their first dietary exchange period. ^d^ n = 21. **^e^** n = 27. ^f^ n = 20. ^g^ n = 25. ^h^ Data were log transformed. | | | |
